# Validity of WHO’s near-miss approach in a high maternal mortality setting

**DOI:** 10.1371/journal.pone.0217135

**Published:** 2019-05-16

**Authors:** Tanneke Herklots, Lieke van Acht, Rashid Saleh Khamis, Tarek Meguid, Arie Franx, Benoit Jacod

**Affiliations:** 1 Division of Woman and Baby, University Medical Centre Utrecht, Utrecht, The Netherlands; 2 Department of Obstetrics and Gynaecology, Mnazi Mmoja Hospital, Stone Town, Zanzibar, United Republic of Tanzania; 3 School of Health & Medical Sciences, State University of Zanzibar (SUZA), Zanzibar, United Republic of Tanzania; 4 Department Obstetrics & Gynaecology, Radboud University Medical Centre, Nijmegen, The Netherlands; University of Michigan Medical School, UNITED STATES

## Abstract

**Objective:**

To evaluate the validity of WHO’s near-miss approach in a low-resource, high maternal mortality setting.

**Design:**

Prospective cohort study.

**Setting:**

Mnazi Mmoja Hospital, the main referral hospital of Zanzibar, Tanzania, from 1 April 2017 until 31 December 2018.

**Population:**

All women, pregnant or until 42 days after the end of pregnancy, admitted at Mnazi Mmoja Hospital, the tertiary referral hospital in Zanzibar.

**Methods:**

Cases of maternal morbidity and mortality were evaluated according to WHO’s near-miss approach. The approach’s performance was determined by calculating its accuracy through sensitivity, specificity and positive and negative likelihood ratios. The approach’s validity was assessed with Pearson’s correlation coefficient between the number of organ dysfunction markers and risk of mortality.

**Main outcomes measures:**

Correlation between number of organ dysfunction markers and risk of mortality, sensitivity and specificity.

**Results:**

26,842 women were included. There were 335 with a severe maternal outcome: 256 maternal near-miss cases and 79 maternal deaths. No signs of organ dysfunction were documented in only 4 of the 79 cases of maternal death. The number of organ dysfunction markers was highly correlated to the risk of mortality with Pearson’s correlation coefficient of 0.89.

**Conclusions:**

WHO’s near-miss approach adequately identifies women at high risk of maternal mortality in Zanzibar’s referral hospital. There is a strong correlation between the number of markers of organ dysfunction and mortality risk.

## Introduction

With globally declining maternal mortality rates (MMR), assessment of severe maternal morbidity is increasingly important in addition to maternal death reviews to evaluate the quality of maternal health care. This has led to reviews and audits of maternal morbidity in not only middle- and high-income countries, where mortality rates are low, but also at facility-level in low-income settings where, despite high morbidity and mortality rates, maternal deaths are relatively rare events. Severe maternal morbidity cases should, however, be selected in such a way as to reflect the same processes as maternal mortality cases. That assumption is not straightforward because causes of severe maternal morbidity are not necessarily those leading to maternal death [1]. In order to draw valid conclusions from an audit process combining maternal morbidity and mortality, one should therefore select cases in which the woman nearly died, designated by the term maternal “near-misses” [2]. The definition of near-misses however intuitive is not unequivocal in practice [3–5]. This led the World Health Organization (WHO) to propose a definition using markers of organ failure, based on scoring systems used in intensive care medicine: the WHO near-miss approach [4]. In practice, this approach uses a two-step system in which women potentially at risk of dying are identified first through disease- and management-based criteria. This is followed by identification of women within this group that are really at risk of dying based on the occurrence of markers of organ failure [2,4]. The final clinical outcome for women in these group is either near-miss—those who survived—or death.

The validity of this approach has been tested in a Brazilian setting with a maternal mortality rate of 170 deaths per 100,000 live births; the number of markers of organ failure appeared to correlate well with the risk of death [6]. Its applicability to settings with higher mortality rates has been established in a large multinational study [7] and several single facility studies [8–10]. However, its validity has never been confirmed in high mortality settings where limited access to diagnostic tools could hamper its validity.

The objective of the current study is to evaluate the validity of WHO’s near-miss approach in a high mortality, low-resource setting by assessing the correlation between the mortality risk and the number of organ dysfunction markers as well as evaluating the number of maternal mortality cases missed by this approach. Secondly, we will compare the type of organ dysfunctions observed in morbidity and mortality cases to test the underlying assumption of a common pathway.

## Methods

### Setting

Zanzibar is an East African archipelago, semi-autonomous to Tanzania and Mnazi Mmoja Hospital (MMH) is its referral hospital. The Obstetrics & Gynaecology (O&G) department of MMH accommodates 11,000 to 13,000 deliveries per year and on average one maternal death occurs per week. Health care services such as essential obstetrical interventions and an intensive care unit (ICU) are generally available and accessible, although not consistently of good quality. The in-hospital maternal mortality ratio (IH-MMR) is above 400 per 100,000 live births [11].

### Study design

Data collection for this study was performed under two research projects (reference numbers ZAMREC/0001/AUGUST/005 and ZAMREC/001/JAN/17) approved by Zanzibar’s Medical Ethical Research Committee. Informed consent was waived because the study concerned only an analysis of clinical files with aggregated, anonymous outcomes.

All cases of maternal near miss and maternal mortality were prospectively identified amongst all women, pregnant or within 42 days after end of pregnancy, admitted to MMH between 1 April 2017 and 31 December 2018. Department-level data on the number of deliveries and live births were extracted from the hospital’s records. MNM cases were identified during the O&G department’s daily clinical staff meetings in which all severe cases were discussed. Simultaneously with this study, a maternal near-miss audit study was performed, contributing to improved case identification and awareness of maternal morbidity within the department. When a patient fulfilled a near-miss criterion, she was actively followed-up by one of the research assistants and the clinical staff. Throughout the entire study period, maternal death cases were identified soon after their occurrence. Maternal death reviews were conducted within 72 hours of the event by the local maternal death review committee consisting of medical doctors of the O&G department, nurse-midwives, an anaesthetist, health workers from the laboratory and blood bank and doctors and nurses from the ICU. Medical files of maternal near-misses and death, when retrieved, were saved, separate from the other files. Relevant information from the patient file, including socio-demographic characteristic, basic obstetric history, a summary of admission, and perinatal and maternal outcomes, was coded, anonymized, stored in a Microsoft Excel spreadsheet and backed-up, accessible to the study team members with a password.

Classification of MNM and mortality cases followed WHO’s near-miss approach. A patient in life-threatening condition was identified following the approach’s set of clinical, laboratory or management-based markers for organ dysfunction, adapted to local availability, as indicated in Table 1. The maternal outcome for these patients was either near-miss or death. The present markers were added to the individual case data in the beforementioned spreadsheet.

**Table 1:** Markers for organ dysfunction, identifying cases in life-threatening condition.

Type of organ dysfunction	Markers (clinical signs, laboratory markers and management actions)
**Cardiovascular**	Shock^1^, use of continuous vasoactive drugs, cardiac arrest, cardio-pulmonary resuscitation, severe hypoperfusion (lactate >5 mmol/L or >45mg/dL)^2^ or severe acidosis (pH <7.1)^2^
**Respiratory**	Acute cyanosis, gasping^3^, severe tachypnea (respiratory rate >40bpm), severe bradypnea (respiratory rate <6bpm), severe hypoxemia (PAO2/FiO2 <200mmHg^2^ or O2 saturation <90% for ≥60min) or intubation and ventilation not related to anaesthesia
**Renal**	Oliguria^4^ non-responsive to fluids or diuretics, dialysis for acute renal failure^5^ or severe acute azotaemia (creatinine ≥300umol/ml or ≥3.5mg/dL)
**Coagulation / haematological**	Clotting failure^6^, use of continuous vasoactive drugs^7^, massive transfusion of blood or red cells (≥5 units)^8^ or severe acute thrombocytopenia (<50,000 platelets/ml)
**Hepatic**	Jaundice in the presence of pre-eclampsia^9^, severe acute hyperbilirubinemia (bilirubin >100umol/L or >6.0mg/dL)
**Neurological**	Prolonged unconsciousness (lasting >12 hours)/coma^10^, stroke^11^, status epilepticus^12^, uncontrollable fits/total paralysis
**Uterine**	Hysterectomy following haemorrhage or infection

Unless otherwise stated, the criteria are reproduced from Say et al. [4]

1) Shock is a persistent severe hypotension, defined as a systolic blood pressure <90 mmHg for ≥60 minutes with a pulse rate at least 120 despite aggressive fluid replacement (>2l)

2) Laboratory test or management intervention that is not available at MMH

3) Gasping is a terminal respiratory pattern and the breath is convulsively and audibly caught

4) Oliguria is defined as a urinary output <30 ml/hr for 4 hours or <400 ml/24 hr

5) Dialysis services have been available in MMH since 26 May 2017

6) Clotting failure can be assessed by the bedside clotting test or absence of clotting from the intravenous site after 7–10 minutes

7) For instance, continuous use of any dose of dopamine, epinephrine or norepinephrine

8) In MMH extended to include all types of blood products and cases in which 5 or more units were requested but not given due to shortage

9) Pre-eclampsia is defined as the presence of hypertension associated with proteinuria. Hypertension is defined as a blood pressure of at least 140 mmHg (systolic) or at least 90 mmHg (diastolic) on at least two occasions and at least 4–6 h apart after the 20th week of gestation in women known to be normotensive beforehand. In MMH, proteinuria is defined as ≥2+ protein on dipstick.

10) Loss of consciousness is a profound alteration of mental state that involves complete or near-complete lack of responsiveness to external stimuli. It is defined as a Coma Glasgow Scale <10 (moderate or severe coma).

11) Stroke is a neurological deficit of cerebrovascular cause that persists beyond 24 hours or is interrupted by death within 24 hours

Condition in which the brain is in a state of continuous seizure

Guided by previous research experience in MMH’s O&G department, we anticipated issues of missing data and poor quality of data. Measures were taken to minimize the influence hereof, such as close collaboration of the researchers and research assistants with the department’s nurses, daily physical presence of the data collectors and weekly updates of data storage. Next to this, ongoing initiatives in the O&G department, in addition to the daily clinical staff meetings, also aimed at improvement of qualitative documentation in patient files. Amongst these projects were the full-time dedication of a registrar to department-level data and the beforementioned maternal near-miss audits. Nevertheless, poor documentation remained an issue.

Data collection was performed by junior investigators (TH, LA, RSK) and seven research assistants throughout the study period, none of whom were involved in patient care. Each research assistant worked on the study for a period of at least 12 weeks and was trained by their predecessor. Training and data collection were supervised by TH and TM. Data quality checks were performed weekly by TH in the spreadsheet and through discussion with the research assistants face to face or, in case of absence from the study site, over telephone. BJ performed additional checks on consistency and quality of the data.

### Data analysis

Incidences and ratios of maternal outcomes were assessed with descriptive statistics. These outcome measures are defined in S1 Table. The distribution of dysfunction of the different organ systems was assessed with descriptive statistics in the group of women with severe maternal outcomes (SMO), including both MNM and MD, and separately for the MNM and MD groups. Relative risk ratios (RR) with a 95% confidence interval were calculated to compare the incidence of a specific type of organ dysfunction in MNM and MD cases.

To analyse the performance of the set of WHO near-miss criteria in this setting, its sensitivity, specificity, positive likelihood ratio and negative likelihood ratio were calculated, given an estimation of its accuracy. Performance in this is defined as the set of criteria’s ability to detect those patients with life-threatening, organ function-violating morbidity. The calculations were performed in the group of only the patients with severe complications.

To analyse the validity of the WHO near-miss approach in the study setting, the number of organ dysfunction markers has been plotted against the mortality risk and their relation has been evaluated using Pearson’s correlation coefficient.

## Results

During the 21-months study period, 26,842 women admitted at MMH–pregnant or within 42 days after end of pregnancy–were included. There were 22,054 deliveries and 22,011 recorded live births (including all live births in cases of multiple pregnancies), 335 (1.3%) women had a severe maternal outcome (SMO), with 256 (1.0%) maternal near-misses and 79 (0.3%) maternal deaths. Maternal outcomes are further detailed in Table 2. The in-hospital maternal mortality ratio (MMR) was 359 per 100,000 live births. A high mortality index (MI) of 0.24 was found, indicating that of all women undergoing very severe morbidity, close to one quarter had died.

**Table 2:** Overview of maternal outcomes.

Maternal outcomes	N (% of total^1^)
Severe maternal outcome cases	335 (1.3)
Maternal near-miss cases	256 (1.0)
Maternal death cases	79 (0.3)
**Overall severe morbidity, near-miss and mortality indicators**	
Severe maternal outcome ratio (per 1000 live births)	15.2
Maternal near-miss incidence ratio (per 1000 live births)	11.6
Maternal near-miss mortality ratio	3.2
In-hospital maternal mortality ratio (per 100,000 live births)	359
Mortality index (MD/SMO)	0.24

1) total = 26,842 included women

The distribution of organ dysfunction criteria found in women experiencing a life-threatening condition is shown in Table 3. Cardiovascular and coagulation organ dysfunction were most prevalent in all women with SMO, 46% and 52%, respectively. Signs of organ dysfunction of the cardiovascular, respiratory, neurological, hepatic and renal systems were significantly more likely to be found in MD than in MNM cases. The highest relative risk ratios were found for respiratory and cardiovascular organ dysfunction, with 5.21 (95% CI 3.34–8.14) and 5.08 (95% CI 3.02–8.53), respectively. By contrast, coagulation disorders and uterine dysfunction were less prevalent in MD cases compared to MNM, with risk ratios of 0.34 (95% CI 0.22–0.53) and 0.58 (95% CI 0.35–0.97), respectively.

**Table 3:** Frequency of organ dysfunction in cases with a severe maternal outcome.

Type of organ dysfunction	SMO (N = 335) % (N)	MNM (N = 256) % (N)	MD (N = 79) % (N)	MD vs. MNM RR (95% CI)
Cardiovascular dysfunction	46 (153)	35 (89)	81 (64)	5.08 (3.02–8.53)
Respiratory dysfunction	35 (116)	23 (58)	73 (58)	5.21 (3.34–8.14)
Coagulation or haematological dysfunction	52 (173)	59 (152)	27 (21)	0.34 (0.22–0.53)
Uterine dysfunction or hysterectomy	29 (96)	32 (81)	19 (15)	0.58 (0.35–0.97)
Neurological dysfunction	11 (36)	4 (11)	32 (25)	3.85 (2.78–5.32)
Hepatic dysfunction	3 (10)	3 (1)	9 (7)	3.97 (2.85–5.54)
Renal dysfunction	17 (56)	13 (32)	30 (24)	2.17 (1.48–3.19)
Any organ dysfunction	99 (331)	100 (256)	95 (75)	

Markers of organ dysfunction were not documented in only 4 out of 79 maternal deaths (5.0%). Adding other criteria such as eclampsia, uterine rupture or sepsis and/or modifying transfusion thresholds further than already performed as suggested by others [12–15] does not, therefore, improve the predictive accuracy of the model. Overall, as outlined in Table 4, the near-miss criteria’s accuracy in identifying women with life-threatening conditions was found to be high.

**Table 4:** Accuracy of the WHO set of severity markers in the prediction of maternal deaths.

		All women, N = 26,842	
		Maternal deaths	
		+	─
**Any WHO criterion**	+	75	256^1^
	─	4	26,507
Sensitivity (95% CI) (%)		94.9 (87.5–98.6)	
Specificity (95% CI) (%)		99.0 (98.9–99.2)	
Positive likelihood ratio (95% CI)		99.3 (87.0–113.3)	
Negative likelihood ratio (95% CI)		0.05 (0.02–0.13)	

1) The maternal near-misses in these calculations are considered the false positives

**Fig 1:**
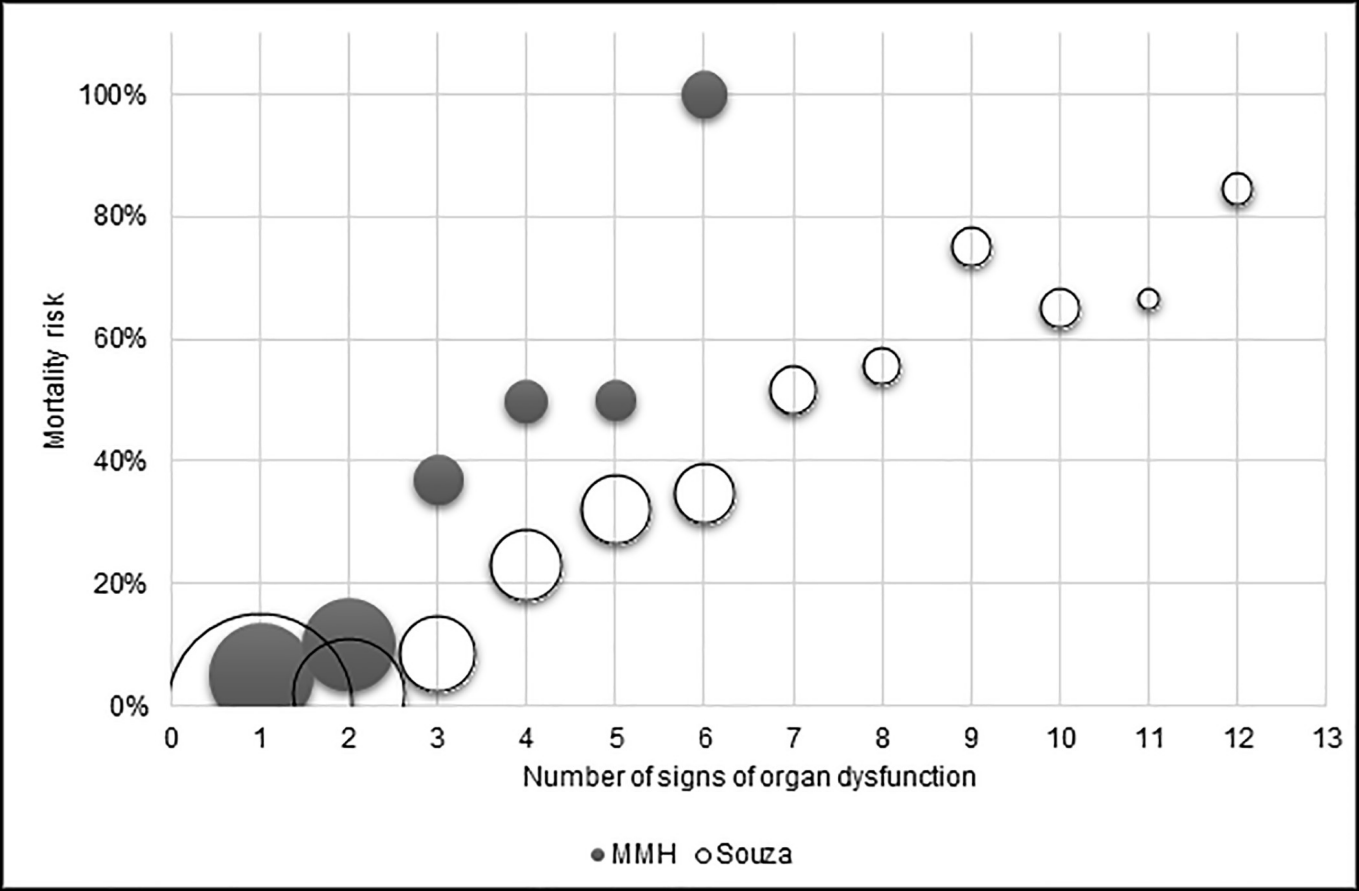
Association between number of severity markers and mortality risk in Zanzibar’s referral hospital (filled circles) and in multiple centres in Brazil aggregated (open circles) [6]. The circles’ diameter is proportional to the number of cases.

Fig 1 shows the relation between the number of markers of organ dysfunction and the risk of mortality. A number of signs of organ dysfunction of six or more (25 of 335) was associated with a 100% risk of mortality. We found a correlation coefficient between the number of severity markers and the risk of death of 0.89, similar to the coefficient of 0.96 found in the seminal study in Brazil [6]. The relation between mortality risk and number of severity markers seems roughly linear in both settings. The main difference lies in the slope of the curve, which is steeper in this study. Women in Zanzibar’s referral hospital have an increased mortality risk even with a low number of severity markers: 5% of women with one marker of organ dysfunction will die. This risk further increases with increasing number of severity markers.

## Discussion

### Main findings

This study shows a strong correlation between the number of organ dysfunction markers and the risk of mortality, validating its use in low-resource, high-mortality settings. The WHO near-miss approach proves to be applicable in this setting with only 5.0% (4 out of 79) of maternal death cases without documentation of organ dysfunction markers.

### Strengths and limitations

To our knowledge, this is the first study to analyse the correlation between markers of organ dysfunction and mortality risk since the seminal study in Brazil. As such, it is the first to validate WHO’s near-miss approach in a high mortality setting. Strengths of our study are its prospective character and the large cohort size enabling the results to even out random variations in the incidence of rare outcomes. The main limitations are the relatively poor quality of documentation in clinical files and the unavailability of a number of WHO near-miss criteria. This has surely caused underestimation of markers of organ dysfunction and might partly explain the difference in the slope of the correlation between number of organ dysfunctions and mortality risk between Zanzibar and Brazil.

### Interpretation

This study demonstrates the validity of WHO’s near-miss approach in a high maternal mortality context. First, the criteria proved to have a high accuracy in identifying patients with life-threatening disorders at serious risk of dying. Second, the number of signs of organ dysfunction is strongly positively correlated to the mortality risk, comparable to that observed in the seminal study in Brazil [6]. Third, the number of women who died without documented signs of organ dysfunction is low, even in this setting where poor documentation is a major issue. Those cases were all four of women who arrived to MMH in near-death condition and there was no to little diagnostics and treatment, nor documentation, performed.

As expected in a high maternal mortality, low-resource setting, the mortality risk increases much more quickly with the number of organ dysfunction markers, compared to a moderate or low maternal mortality setting. A patient with one sign of organ dysfunction has a risk of mortality of 5%, one with two signs a risk of 10%. A comparable level of mortality risk is only reached in patients with three markers of organ dysfunction in Brazil. At least part of this distinction reflects the difference in quality of care between the Brazilian centres and MMH. This suggests that women in high-mortality settings do not die (solely) because their condition is more severe than elsewhere but because of sub-optimal quality of care.

In assessing cases of patients in life-threatening condition, underestimation likely has played a role as organ dysfunction markers are not measured comprehensively due to limited diagnostic and therapeutic possibilities in MMH. This is mostly reflected in the incidence of laboratory-based criteria, which is lower in high-mortality, low-resource settings, including this study’s, compared to moderate mortality settings, as shown in a large multi-country study [7]. Incomplete patient file documentation by health workers as well as a poor file-keeping infrastructure in MMH also contribute.

As expected, there is a difference in types of organ dysfunction between patients surviving and those dying in this cohort. The latter are more likely to have suffered from cardiovascular and/or respiratory collapse while the former are more likely to have experienced haematogical/coagulation dysfunction and uterine dysfunction. These findings are comparable to those found by others [1,7,8]. This distinction is insightful, showing that, despite identifying patients in life-threatening condition through the same set of criteria, the ones that survive are often on different pathways than the ones that die, and vice versa. This, firstly, encourages more research into the various characteristics within the group of severely ill patients, including sociodemographic, pregnancy-related, patient’s behaviour-related and received care-related characteristics. Secondly, it can inform policy-making for resource allocation, promoting the necessary diagnostic and management skills and tools, e.g. organ function laboratory test, CPR training and blood product availability.

Including severe morbidity next to mortality improves the assessment of a setting’s quality of maternal health care, going beyond the long-used indicator MMR. Doing this with a selected approach, that allows for local adjustment, enables comparability between settings and over time, which is relevant to making, implementing and reviewing policies for quality improvement. The mortality index allows to correct for the severity of the population studied because, irrespective of the background population case-mix, it puts the maternal death rate in perspective to the only group at significant risk of dying. As such, it provides a more accurate measure of the impact of clinical care than when using the maternal mortality rate alone, provided that near-miss cases are selected following well-defined, widely applicable, though locally-adjusted criteria.

The robustness of any conclusion on the quality of care is dependent on the quality of the data collected and challenges in this should be acknowledged and addressed in maternal morbidity registration. We recommend adhering to WHO’s near-miss criteria, adjusted to the specific setting as needed, because it enables meaningful comparison between similar reference populations. We show in our study that it is applicable and valid also in low-resource, high-mortality settings.

## Conclusion

WHO’s near-miss approach adequately identifies women at high risk of maternal mortality in Zanzibar’s referral hospital. There is a strong correlation between the number of markers of organ dysfunction and mortality risk while only very few mortality cases do not fulfil any WHO criteria. The mortality index is an appropriate measure of quality of maternal health care complementing mortality rates.

## Supporting information

S1 Table Outcome measures. (DOCX)

S1 Database (XLSX)
