# Supplementary material for: Validity of WHO’s near-miss approach in a high maternal mortality setting
Source: PLoS One. 2019 May 16;14(5):e0217135. doi: 10.1371/journal.pone.0217135 (PMC6522045; doi:10.1371/journal.pone.0217135)
Supplement: S1 Table — (DOCX) [file pone.0217135.s001.docx]

**S1 Table**: Outcome measures

| **Measure** | **Description** |
| --- | --- |
| **Severe maternal outcome (SMO) ratio** | Number of cases of maternal near-miss and maternal death per 1000 live births |
| **Maternal near-miss (MNM) incidence ratio** | Number of cases of maternal near-miss per 1000 live births |
| **Maternal near-miss mortality ratio** | Ratio between number of maternal near-miss cases and number of maternal deaths |
| **Maternal mortality ratio (MMR)** | Number of maternal death cases per 100,000 live births |
| **Observed mortality index (MI)** | Number of maternal death cases divided by the total number of cases with a severe maternal outcome |
